# Establishing a Framework for Fused Filament Fabrication Process Optimization: A Case Study with PLA Filaments

**DOI:** 10.3390/polym15081945

**Published:** 2023-04-19

**Authors:** Jack Grubbs, Bryer C. Sousa, Danielle L. Cote

**Affiliations:** Department of Mechanical and Materials Engineering, Worcester Polytechnic Institute, Worcester, MA 01609, USA; jagrubbs@wpi.edu (J.G.); bcsousa@wpi.edu (B.C.S.)

**Keywords:** additive manufacturing, FFF, PLA, mechanical properties, dimensional accuracy, optimization

## Abstract

Developments in polymer 3D printing (3DP) technologies have expanded their scope beyond the rapid prototyping space into other high-value markets, including the consumer sector. Processes such as fused filament fabrication (FFF) are capable of quickly producing complex, low-cost components using a wide variety of material types, such as polylactic acid (PLA). However, FFF has seen limited scalability in functional part production partly due to the difficulty of process optimization with its complex parameter space, including material type, filament characteristics, printer conditions, and “slicer” software settings. Therefore, the aim of this study is to establish a multi-step process optimization methodology—from printer calibration to “slicer” setting adjustments to post-processing—to make FFF more accessible across material types, using PLA as a case study. The results showed filament-specific deviations in optimal print conditions, where part dimensions and tensile properties varied depending on the combination of nozzle temperature, print bed conditions, infill settings, and annealing condition. By implementing the filament-specific optimization framework established in this study beyond the scope of PLA, more efficient processing of new materials will be possible for enhanced applicability of FFF in the 3DP field.

## 1. Introduction

### 1.1. Additive Manufacturing

Additive manufacturing, commonly referred to as 3DP, is a manufacturing approach that has matured substantially over the past several decades, with technologies spanning several industries capable of processing a wide variety of material types. Originating as a polymer processing technique, 3D printers deposit material in selective regions of a build plate in a layer-by-layer fashion according to a prescribed part geometry generated from a 3D computer-aided design file [1,2]. Several types of polymer 3DP technologies exist, which can be categorized by their feedstock material form as resin, powder, or filament. Resin-based 3DP processes, such as stereolithography, leverage photosensitive resins that can be cured using high-energy ultraviolet light. Resin curing processes typically print parts with high build rates and are seen as some of the most precise 3DP processes with high geometrical resolution at the micron scale. However, material options are restricted to solely curable resins, and high dimensional accuracy comes at the cost of limited printable part volume [3,4,5]. Powder-based polymer 3DP processes, such as selective laser sintering and binder jetting, rely upon the fusion or sintering of polymer particles through melting or chemical bonding to form a bulk component. These processes can produce near-net-shape parts with customizable microstructures, including multi-material and functionally graded prints. However, apart from the high material cost of polymer powders, elevated levels of porosity are common in the final components, making post-processing a necessary step for structural applications [3,5,6]. Finally, filament 3DP processes, such as FFF, extrude thermoplastic-based filaments through a heated nozzle onto a build plate. Filament extrusion 3DP is typically low cost, allows for multi-material prints, and requires minimal equipment maintenance. However, relatively high printed layer thickness results in issues with surface quality, and processing parameter optimization can be complex given the number of tunable inputs [7,8,9]. Many polymer 3DP systems are commercially available, yet FFF is the most commonly used processing technique, particularly in the consumer sector, due to its ease of use and enhanced selection of processable materials [10,11]; as such, FFF will serve as the focus of this study.

### 1.2. FFF: Process Overview and Processing Parameters

As alluded to previously, FFF is an extrusion-based 3DP process whereby a thermoplastic filament is passed through a heated nozzle to create a layer of a specified geometry. After printing the first layer on the print bed, subsequent layers may be extruded and bonded to the previous layer, facilitated by the semi-molten state of the extruded filament. Depending on the part geometry, a removable support material may be used, which is typical with overhanging sections, such as holes, arches, or bridges [12]. FFF is particularly versatile regarding material compatibility, spanning many polymer types and filament colors, with the principal requirement being that filaments must be extrudable through a heated nozzle. The most common filaments used in FFF are acrylonitrile butadiene styrene (ABS) and polylactic acid (PLA) due to material availability and relative ease of printability; however, several other filament types are also used, including nylon, polycarbonate (PC), polyethylene terephthalate glycol (PETG), thermoplastic polyurethane (TPU), and polyether ether ketone (PEEK) [13,14,15,16]. Composite blends are also utilized as FFF filaments, with reinforcement phases ranging from carbon fibers to wood fillers, for a variety of functional and esthetic applications [17,18,19,20].

Many processing parameters can be manipulated in a “slicer” software, which generates the “g-code” to control printer operations, prior to printing in conventional FFF systems to accommodate the desired part design and material type. These parameters can be categorized into four groups: (1) print bed parameters, (2) nozzle parameters, (3) path configuration parameters, and (4) part-specific parameters.

The print bed parameters often manipulated are the bed temperature and the bed surface, both of which serve to increase first layer adhesion, which can dramatically increase the overall part quality, properties, and behavior [21]. Print beds can remain unheated depending on the polymer type, such as with PLA. However, heated beds can minimize void formation in initially printed layers near the print bed, as well as reduce the probability of part distortion and shrinkage by minimizing thermal gradients between the nozzle and the print surface [22,23]. Usable print bed temperatures range widely across material types—varying in experiments found in the literature from 20 to 105 °C for PLA and its composites and from 40 to 110 °C for ABS—and are usually set at values at or above the glass transition temperature (T_g_) [23,24,25,26,27]. Bed surface conditions are commonly adjusted based upon user preference, but they can be critical to manipulate for difficult-to-print filaments, such as ABS [23,28]. Typical modifications to the original print surface, which are usually glass or polymer in conventional FFF printers, include the addition of roughened surfaces (such as films or tape) or chemical coatings (such as hairspray or glue) [29]. 

Adjusting nozzle parameters provides another source for process optimization, where nozzle size, nozzle temperature, and material feed rate can all be customized for a given print and filament. Nozzle temperature is arguably the most crucial setting to fine tune, as the entirety of FFF relies upon adequate melting of the thermoplastic filament so that extrusion through the nozzle is possible. Adequate polymer viscosities are targeted through nozzle temperature adjustments so that proper filament fluidity is achieved, allowing for repeatable and controllable polymer beads to be fabricated [8]. Nozzle temperatures also vary depending on the filament type—from 180 to 230 °C for PLA, from 210 to 250 °C for ABS, and from 210 to 240 °C for PETG—typically at values above the given polymer’s melting temperature (T_m_) [27,30,31]. Filament feed rates may be modified to further tailor the degree of material extrusion, especially when coupled with path configuration parameters, such as traverse speed and acceleration. Proper extrusion setting selection through modifying these parameters allows for tight control of layer thickness, enhanced surface quality, and minimal residual stresses [8]. 

Finally, part-specific parameter adjustments give FFF users the flexibility to target a large window of part properties and surface features by varying parameters such as wall thickness, infill density, and infill pattern [8,32,33]. Fine-tuned wall thickness values can significantly minimize surface roughness, while tailored infill density and infill pattern have resulted in higher strength-to-weight ratios, lowered print times, and reduced material costs [34,35,36]. Other processing decisions beyond those related to the parameters mentioned above, such as the selection of build orientation relative to the build plate, must be made and can significantly impact part properties [37]. 

### 1.3. PLA: Overview and FFF Exploration

Given the relevance of PLA and its composites to industrial and prototyping applications, PLA filaments are explored in this study. PLA is a biodegradable thermoplastic material commonly derived from renewable resources, such as corn and rice [13,38]. The molecular weight and stereochemical composition of PLA are determined by the nature of polymerization, which usually occurs through monomer synthesis, polycondensation, ring-opening polymerization, and azeotropic dehydration condensation [39,40]. PLA’s thermal characteristics are quite sensitive to its production methods and stereoisomeric form, which is usually L-lactide, D-lactide, or meso-lactide form. The T_g_ and T_m_ for semi-crystalline PLA typically reside around 60 and 180 °C, respectively. Notable shifts in T_g_, T_m_, and percent crystallinity have been observed as PLA’s chemical structure changes and stereoisomeric defects are introduced, usually to lower values as the molecular weight and D-lactide content increase [39,40]. PLA follows typical rheological transition from Newtonian behavior to non-Newtonian behavior at increasing shear rate (around 10 s^−1^), which is highly sensitive to molecular weight, polymer branching, composition, and crystallinity [40,41,42]. The high nozzle temperatures, low viscosity at high shear rates through the nozzle, and high melt stability all make PLA an excellent candidate for FFF printing from a thermal perspective.

PLA specimen production through FFF has been explored quite extensively in recent years. Rodríguez-Panes et al. highlighted the role of layer height, infill density, and layer orientation on the mechanical properties of PLA prints compared to ABS prints; increasing infill density led to dramatic improvements in strength and ductility, whereas decreasing layer height and aligning layer paths parallel to the build direction also moderately improved strength [43]. Yadav et al. printed PLA samples for compression testing with varying infill densities and patterns, noting an increase in ultimate compressive strength with increasing infill density and changes in infill pattern [44]. Fatigue properties of 3D-printed PLA specimens were also explored under a tension–compression loading regime, whereby Afrose et al. determined that modifications to the infill orientation relative to the testing direction—specifically, at 0°, 45°, and 90° relative to the testing direction—significantly impacted fatigue life, with optimal performance at a 45° infill [45]. 

Apart from prints with pure PLA, filament additives, notably from composite reinforcements, have also been explored in recent FFF process development efforts. Cuiffo et al. investigated the use of calcium carbonate-based additives in PLA, while Khoo et al. and Heidari-Rarani et al. researched the influence of nanocellulose and continuous carbon fibers in PLA, respectively, for FFF applications; these researchers highlighted the chemical and physical ramifications involved with PLA composite filament printing in FFF, such as changes in thermal characteristics and mechanical properties, with potential for part property enhancement compared to virgin PLA [46,47]. However, FFF process optimization efforts become complicated when thermophysical filament properties change, even from contributions beyond those associated with composite additives. Alterations in filament and 3D-printed material characteristics and behavior have been observed in virgin polymer materials with only modifications to color or manufacturer. Valerga et al. studied the influence of polymer pigmentation (or color) on dimensional accuracy and surface quality at varying extrusion temperatures. The addition of pigments appeared to increase deviations from nominal dimensions and decrease part surface quality from increased melt fluidity [48]. Katalinic et al. also studied the mechanical performance of PLA tensile specimens printed from 14 different colored filaments, noting that stiffness, strength, and toughness metrics all fluctuated with the feedstock material’s color changes [49]. Changing filament manufacturer leads to a more anticipated change in printed part properties, given the variations in thermal histories, polymer compositions, etc., from filament fabrication; this was noted by Khabia et al. through the mechanical testing of printed ABS specimens from filaments of variable quality, where notable increases in specimen toughness were observed with higher-quality filaments [50]. 

Post-processing treatments, such as annealing, have been incorporated into 3DP routines to further tailor part properties for the desired application. Annealing treatments are typically performed at slow heating and cooling rates between the T_g_ and T_m_ for semi-crystalline polymer samples [51]. When treatment conditions are selected properly, residual stresses from fast cooling can be relieved and crystallinity levels increase, influencing optical and mechanical properties [51,52,53,54]. Wach et al. achieved improvements in flexural stress values by 11–17% when annealing FFF-printed PLA specimens to upwards of 30 °C above the measured T_g_ values, whereas Szust et al. increased part strength by 24% after annealing at 60 °C for 1 h [55,56]. However, loss of property control, part shrinkage, and decreased ductility often accompany positive material strength changes after annealing, warranting user caution when implementing this technique as a post-3DP tool [51,57]. With variations in filament characteristics adding another degree of complexity to processing parameter optimization efforts, careful consideration of filament properties during material selection and post-processing operations must be taken; implementing proper material characterization protocols is necessary to achieve predictable processing and desired component specifications.

### 1.4. Aims and Objectives

With a highly dimensional parameter space in FFF printing—including filament type, the four aforementioned processing parameter categories, and post-processing options—the difficulty of processing parameter optimization for a given filament–printer combination serves as a primary obstacle for the scalability of functional part production beyond basic prototyping applications [8]. Dimensional accuracy and tensile properties are among the most important quality metrics for FFF prints; thus, the present analysis focuses on process parameter adjustments with part geometry and material behavior in mind [33]. Many studies in the current literature highlight the importance of specific processing parameters on part characteristics, but they often neglect to detail the interconnectedness of different processing steps, processing parameters, and filament properties and how these fit into an effective process optimization framework for FFF filament-printer combinations. Therefore, the aim of this study is to systematically investigate the effects of frequently manipulated processing parameters and printer conditions on the dimensional accuracy and mechanical properties of 3D-printed PLA tensile specimens. By varying several PLA filament colors and brands, a diverse pool of commercially available material types is processed and characterized. This work serves to aid FFF users by providing a multi-step framework, using PLA as a case study, to guide FFF filament–printer process optimization for functional 3DP applications.

## 2. Materials and Methods

### 2.1. PLA Filaments

Nine commercially available PLA filaments were utilized in this study, which vary by brand and color. The filaments manufactured by OVERTURE (Overture 3D Technologies LLC, Missouri City, TX, USA), SUNLU (SUNLU, Zhuhai, China), and HATCHBOX (HATCHBOX 3D, Pomona, CA, USA) were sourced in white (“White”), black (“Black”), and green (“Green”) colors, all with a standard 1.75 mm diameter. These nine filament variations were specifically chosen to provide diversity in filament manufacturing history and chemical makeup, as determined by the different manufacturers and colors, thereby enabling the identification of disparities in filament mechanical and thermal properties, which may affect print success and part performance. While other filament colors and manufacturers could have been explored for the current development efforts, these filaments serve as a representative and randomized sample of what is readily available on the market that can be sourced for all means of consumer and industrial FFF uses. All filaments were stored in sealed plastic bins with rechargeable silica packets to minimize filament degradation from humidity exposure.

### 2.2. FFF Processing of PLA Filaments

**Figure 1:**
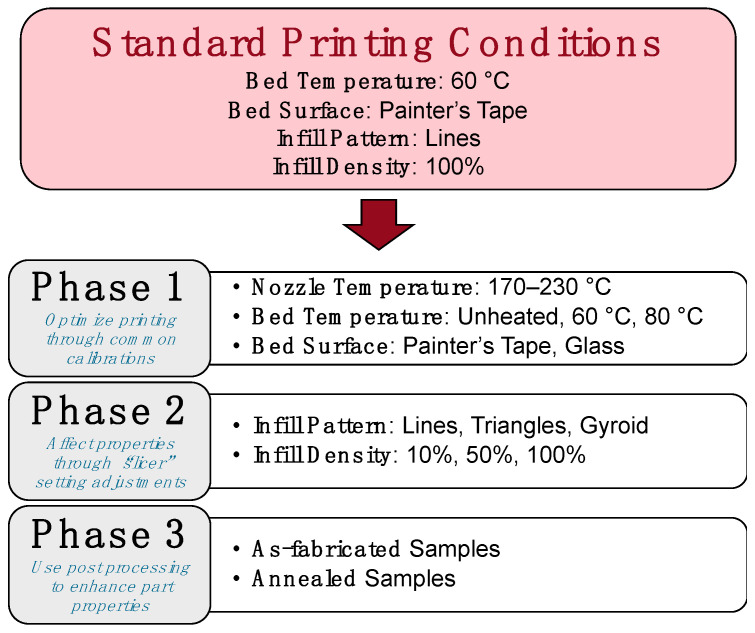
Three-phase experimental setup to demonstrate processing parameter optimization for PLA in FFF.

The present study was conducted in three phases to highlight how specific processing parameter changes throughout the FFF process affect the mechanical properties and dimensional accuracy of PLA specimens produced using different filaments, as detailed in Figure 1. Phase 1 focused on print calibrations, whereby an optimized (or calibrated) parameter set for each filament was determined based on variations from a standard set of conditions for PLA. Specifically, nozzle temperature was first calibrated for each filament using an open-source “temperature tower” design [58], ranging from 170 to 230 °C in order to identify the best extrusion temperature through the heated nozzle. Once calibrated, the bed temperature and bed surface were varied based on the parameters highlighted in Figure 1, leading to four parameter sets for each filament: “Standard” (using the standard conditions), “Unheated” (using an unheated printer bed instead of 60 °C), “80 °C” (using an 80 °C bed temperature instead of 60 °C), and “Glass” (using the glass bed surface instead of painter’s tape at 60 °C). 

Once an optimized parameter set was determined in Phase 1, two important “slicer” settings in Phase 2 were adjusted in the Ultimaker Cura 4.11.0 software (Ultimaker B.V., Utrecht, The Netherlands): infill pattern and infill density. Three infill patterns—Lines, Triangles, and Gyroid—and three infill densities—10%, 50%, and 100%—were combined using the optimal printing conditions for each filament from Phase 1, leading to nine total parameter sets for each filament. An “optimal” and a “worst-case” parameter set were defined for each filament based upon the resulting dimensional accuracy and mechanical properties from Phase 2. These parameter sets were then used in Phase 3, where samples were printed and then annealed to elucidate the effects of post-processing on part properties; the details of annealing are discussed in Section 3.4. 

**Figure 2:**
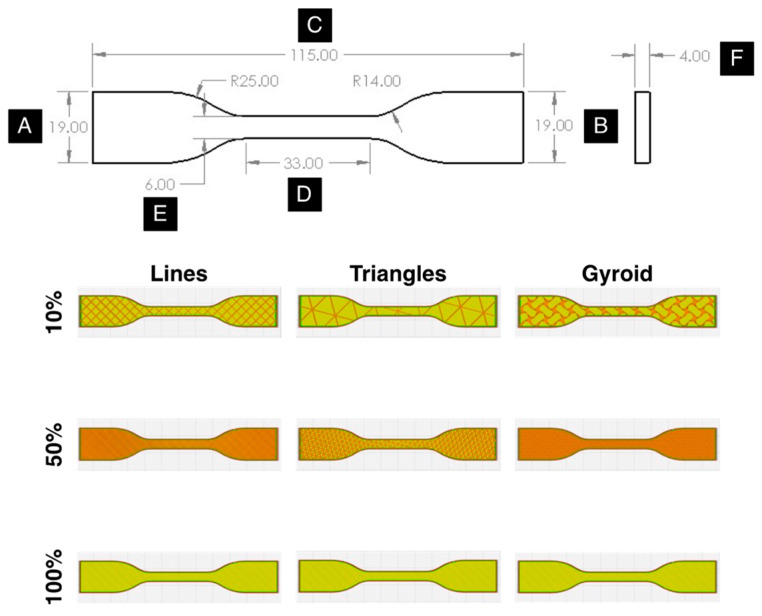
Tensile specimen geometry (with dimensional measurements A through F in mm) based upon the ASTM D638-14 Type IV specifications, alongside specimen cross sections used in Phase 2 for each infill density and infill pattern.

All prints were performed using a Creality CR-10S printer (Creality 3D, Shenzhen, China) equipped with 0.4 mm brass nozzles. For each print, a fresh surface was prepared, either by placing new painter’s tape on the glass bed or by cleaning the glass bed with 90% isopropyl alcohol to remove any residue. The bed was then leveled by adjusting the height of the print bed at each corner so that a piece of standard printer paper could slide between the nozzle and the print bed with moderate friction; this is a fast and commonly used technique to level the bed so material can be properly deposited on the build surface. Each print was composed of three tensile specimens surrounded by a three-layer skirt to ensure proper bed adhesion prior to printing. The samples were printed using a 50 mm/s print speed at a 0.2 mm layer height and 0.8 mm wall thickness (with a wall count of two). The material feed rate (“Flow” in Cura) was set at 100% with retraction enabled at a 5.0 mm retraction distance and 45.0 mm/s retraction speed. The samples’ geometry was based upon the Type IV tensile specimen dimensions detailed in the ASTM Standard D638-14 [59] but scaled down according to the dimensions in Figure 2 to accommodate the tensile testing apparatus’s size limitations. While the samples were expected to be anisotropic in behavior and the ASTM Standard D638-14 specifies five samples must be printed and tested both normal to and parallel with the principal axis of anisotropy [59], only three specimens were printed and tested per set of processing conditions per filament type in a single build orientation due to resource and equipment availability limitations. The testing results of the three specimens were compared to ensure consistency of part geometry and mechanical property evaluations. Figure 2 also highlights a digital cross section of an example tensile specimen from the Cura “slicer” software for Phase 2 printing, whereby the infill settings vary for the different pattern–density combinations.

### 2.3. Approach to Geometric and Mechanical Assessments

Each tensile specimen was assessed geometrically and mechanically after printing. A set of digital calipers with 0.01 mm resolution, ±0.03 mm accuracy, and 0.01 mm repeatability was used to measure the degree to which each sample deviated from the prescribed geometry shown in Figure 2. An average percent deviation for the six dimensions (A through F) across the three specimens per processing condition per filament type was calculated. Similarly, each of the three specimens per processing condition per filament type was subsequently tested in uniaxial tension on a Mecmesin MultiTest 2.5-dVu system (Mecmesin, Slinfold, UK) equipped with a 2500 N load cell and 5000 N wedge grips. Stress and strain calculations were made in the VectorPro 6.10.0.0 software based upon the load and displacement information collected by the system and the input specimen geometry. The stress–strain values for the plotted samples in Section 3.1 are reported in Spreadsheet S1 (“Phase 1 Stress–Strain Curves”) as Supplementary Materials. The average elastic modulus (E), offset yield strength ($\sigma_{y}$), ultimate tensile strength ($\sigma_{UTS}$), and strain at fracture ($\varepsilon_{f}$) values were measured for each sample throughout the three phases of the study; the strength-to-mass ratio ($\frac{\sigma_{UTS}}{m}$) was also utilized in Phase 2 when the infill settings were varied, after measuring each sample’s mass on a Mettler Toledo ML4002T/00 Precision Balance (Mettler Toledo, Columbus, OH, USA). The tabulated values for specimen dimensional accuracy and mechanical properties across Phase 1 through Phase 3 for each filament are reported in Spreadsheet S2 (“Phase 1 Testing Values”), Spreadsheet S3 (“Phase 2 Testing Values”), and Spreadsheet S4 (“Phase 3 Testing Values”) as Supplementary Materials.

To determine the optimal set of processing conditions for a given filament, a simple linear ranking scheme was employed. The goal of this ranking scheme was to identify the parameter set that produced the lowest combined “score” ($S_{Combined}$) for each filament using the part geometry and tensile testing data, while weighing geometrical accuracy and mechanical performance equally. For each filament type, the geometrical measurements and mechanical testing results were compared across processing parameter sets, ranking each dimension (A through F) and mechanical property (E, $\sigma_{y}$, $\sigma_{UTS}$, and $\varepsilon_{f}$, as well as $\frac{\sigma_{UTS}}{m}$ in Phase 2) to determine the optimal parameter set. A rank of “1” was given as the best parameter set when calculating a specific score, which corresponds to the lowest dimensional deviation for the dimension “score” ($S_{Dimension}$) and the highest mechanical property value for the property “score” ($S_{Property}$), with increasing rank as the dimensional accuracy and mechanical properties worsen. As there are different numbers of inputs into the $S_{Dimension}$ and $S_{Property}$ values and it is desired that they both weigh equally to calculate the $S_{Combined}$, a scale factor was used for each score so that the maximum and minimum possible scores are the same for the $S_{Dimension}$ and $S_{Property}$. Therefore, Equations (1)–(3) below were used to produce the $S_{Dimension}$, $S_{Property}$, and $S_{Combined}$ scores for each filament in Phase 1:

(1) $$S_{Dimension}=K_{Dimension}\left(R_{A}+R_{B}+R_{C}+R_{D}+R_{E}+R_{F}\right)$$

(2) $$S_{Property}=K_{Property}\left(R_{E}+R_{\sigma_{y}}+R_{\sigma_{UTS}}+R_{\varepsilon_{f}}\right)$$

(3) $$S_{Combined}=S_{Dimension}+S_{Property}$$

where $K_{Dimension}$ and $K_{Property}$ are the scale factors of 2 and 3, respectively, to yield a maximum and a minimum possible $S_{Dimension}$ or $S_{Property}$ value of 48 and 12, respectively, and $R_{i}$ is the rank of the ith dimension or property, ranging from 1 to 4 in Phase 1 for the four parameter sets tested. A similar scoring regime was utilized in Phase 2, adding the $\frac{\sigma_{UTS}}{m}$ metric to the $S_{Property}$ score from Equation (3), resulting in Equation (4):

(4) $$S_{Property}=K_{Property}\left(R_{E}+R_{\sigma_{y}}+R_{\sigma_{UTS}}+R_{\varepsilon_{f}}+R_{\sigma_{UTS}/m}\right)\;$$

The number of variables in Equation (4) is different from Equation (2), so the scale factors must be adjusted accordingly to achieve the same maximum and minimum possible $S_{Dimension}$ or $S_{Property}$ values. As such, the $K_{Dimension}$ and $K_{Property}$ values were adjusted to be 5 and 6, respectively, yielding a maximum and a minimum possible score of 540 and 60, respectively, for $S_{Dimension}$ and $S_{Property}$. Additionally, since nine parameter sets were varied in Phase 2, $R_{i}$ ranged from 1 to 9. While the optimal parameter set from Phase 1 for a given filament was the set with the lowest $S_{Combined}$, the optimal and worst-case parameter sets in Phase 2 were those corresponding to the lowest and highest $S_{Combined}$ for each filament type, respectively. The filament rankings across different parameter sets in Phases 1 and 2 are also reported in Spreadsheet S5 (“Phase 1 Rankings”) and Spreadsheet S6 (“Phase 2 Rankings”) as Supplementary Materials. It is worth mentioning that the optimal and worst-case parameter sets might be selected differently in Phases 1 and 2 if dimensional accuracy and mechanical properties were weighed unequally; therefore, it is up to 3DP users to define their target metrics and adjust their $K_{Dimension}$ and $K_{Property}$ scale factors accordingly when using this format of ranking. Additionally, if another ranking scheme were used altogether, it would be likely that the optimization results would change, and 3DP users should be aware of this during process optimization.

### 2.4. Thermal Analysis and Annealing

Thermal analysis via differential scanning calorimetry (DSC) was completed on each filament and a selected subset of 3D-printed samples to observe the effects of filament production, filament color, FFF processing, and specimen annealing on the polymers’ thermal characteristics. A TA Instruments Discovery DSC (TA Instruments, New Castle, DE, USA) equipped with nitrogen purge gas was utilized for the PLA thermal analysis. Preliminary calibrations for heat capacity were performed according to the specifications of the ASTM Standard E1269-11 using sapphire [60]. Several first-order and second-order transformations were observed through the heating and cooling runs prescribed by the ASTM Standard E1356-08 [61], namely glass transition, cold crystallization, and melting. The test procedure involved equilibrating a test sample at 25 °C in a TZero Aluminum pan with a TZero Hermetic lid, heating the sample at 10 °C/min to 250 °C, holding the temperature at 250 °C for 10 min, cooling the sample at 20 °C/min until 25 °C was reached, and then equilibrating at 25 °C. This test was performed twice consecutively on a single piece of each filament, allowing for the identification of the thermal characteristics in the first and second runs that were dependent and independent of the material’s processing history, respectively. All samples were run against an empty TZero Aluminum pan with a TZero Hermetic lid as a reference sample. Percent crystallinity (χ) was calculated using Equation (5) based upon [62]:

(5) $$\chi =\frac{\Delta H_{m}-\Delta H_{cc}}{\Delta H_{m}{}^{o}}\times 100\%,$$

where $\Delta H_{m}$ is the enthalpy change associated with the melting of the semi-crystalline PLA (in J/g); $\Delta H_{cc}$ is the enthalpy change associated with the cold crystallization of the semi-crystalline PLA (in J/g); and $\Delta H_{m}{}^{o}$ is the enthalpy change associated with the melting of fully crystalline PLA, which is assumed to be 93.6 J/g [47].

Informed by the DSC thermal analysis, annealing was performed in Phase 3 on the specimens produced by the optimal and worst-case parameter sets from Phase 2 for each filament type. Annealing was completed at 75 °C for 15 min; the selection of annealing temperature and time will be discussed further in Section 3.3. A Thermotron SM-1.0-8200 Benchtop Temperature/Humidity Chamber (Thermotron, Holland, MI, USA) equipped with a Product Temperature Control (PTC) thermocouple was utilized for annealing the 3D-printed PLA samples. Dimensional, thermal, and mechanical property changes were monitored before and after annealing to assess the viability of implementing annealing as a post-processing technique to enhance part performance. The tabulated DSC curve data for all plotted samples are reported in Spreadsheet S7 (“Filament DSC Heating & Cooling Curves”) and Spreadsheet S8 (“Printed Specimen DSC Heating Curves”) as Supplementary Materials.

## 3. Results and Discussion

### 3.1. Phase 1—Print Calibrations

**Figure 3:**
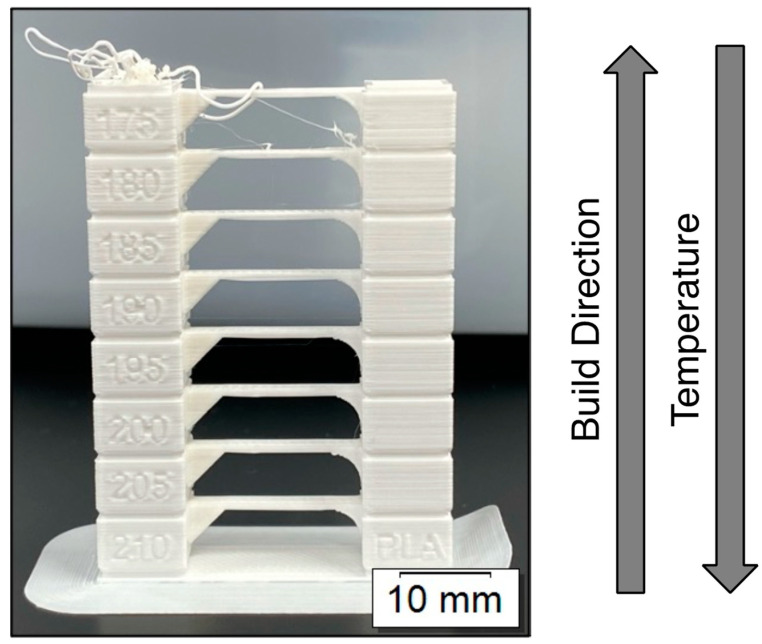
Example temperature tower used in Phase 1 to determine the optimal nozzle temperature for a given filament.

With FFF, preliminary calibration is crucial so that adequate melt conditions can be set at the nozzle and proper bed adhesion can be achieved for a given printer–filament combination. Nozzle temperatures were first calibrated for each filament using temperature towers, as shown in Figure 3. These towers print sections at a specified temperature that bridge from one side to the other, increasing in temperature as it moves vertically from section to section away from the build plate. Feature resolution (e.g., from the inscribed temperature markings), bridging capability, and stringing were all qualitatively assessed to identify build quality at each temperature. The optimal temperature for each filament was identified as the lowest temperature that maintains feature resolutions, enables proper bridging, and contains minimal stringing. 

Table 1 contains the optimal nozzle temperatures for all nine filaments. The White filaments for each brand appear to have optimal temperatures less than or equal to the Black and Green filaments. This may be due to differences in the rheological behavior of the highly pigmented filaments when molten, as well as differences in processing history, which affect melt flow during extrusion through the nozzle [42,48]. Across each filament color, SUNLU typically has the highest optimal nozzle temperature, followed by OVERTURE and then HATCHBOX. While the disparities in temperature here are not drastic, these again may be due to slight differences in compositions, molecular weights, etc., from being manufactured by different companies, thus likely affecting the rheological behavior at higher temperatures and, thus, the ideal extrusion temperature through the nozzle. Interestingly, these nozzle temperatures are all at the upper end of the recommended values from the manufacturers, which are 190–220 °C for OVERTURE, 200–230 °C for SUNLU, and 180–210 °C for HATCHBOX. While small differences are highlighted in Table 1 across brand and color, these results demonstrate that proper steps need to be taken to calibrate nozzle temperatures across filaments on a given printer, as no single temperature can be guaranteed as optimal for a given material type.

After proper nozzle temperatures were selected, Phase 1 tensile specimens were printed in the “Standard”, “Unheated”, “80 °C”, and “Glass” conditions specified in Section 3.2, measured for geometry, and tested under uniaxial tension. After ranking the measurement data and calculating the $S_{Dimension}$ and $S_{Property}$ values using Equations (1) and (2), the scores for each filament are reported in Table 2. Across filament brands and colors, the “Standard” and “Glass” conditions typically produced the best (or lowest) $S_{Dimension}$ value. These parameter sets both had heated print beds at 60 °C, while the “Unheated” and “80 °C” parameter sets had bed temperatures at 25 and 80 °C, respectively. As previously mentioned, the literature findings have demonstrated that, while bed heating is not necessary for PLA, slightly elevated temperatures at or above the T_g_ are ideal to produce prints with proper bed adhesion and minimal distortion upon printing; for PLA, these temperatures often range from 60 to 80 °C, which align with manufacturer recommendations. Between the “Standard” and “Glass” conditions, bed surface preparation with tape versus glass did not seem to make a notable difference on geometrical accuracy. Aligning with the reported literature, the “Unheated” and “80 °C” conditions resulted in the worst dimensional control from part distortion or part drooping, respectively. Part distortion may originate from thermal gradients imposed between the print bed and extruded material from the hot end, while part drooping is likely due to excessive softening of printed layers from higher bed temperatures above the T_g_ [28].

**Figure 4:**
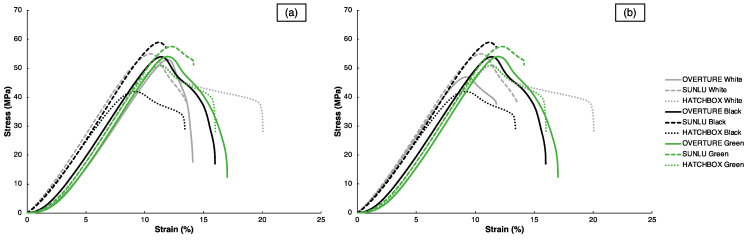
Engineering tensile stress–strain curves for representative Phase 1 samples for each filament that express (**a**) the best mechanical properties and (**b**) the optimal parameter sets.

The “Standard” and “80 °C” conditions generally produced the specimens with the best mechanical properties across all filament types. There was no apparent pattern by which conditions produced the worst mechanical properties, but it was least frequently observed with the “Standard” condition. With FFF specimens, uniaxial tensile properties are severely affected by interlayer defects and voids, particularly those driven by poor layer adhesion [9,63]. With the assistance from the painter’s tape and higher bed temperatures in the “Standard” and “80 °C” conditions, bed adhesion and subsequent layer adhesion were likely enhanced compared to the other two conditions. The engineering stress–strain curves for the representative samples with the best mechanical properties, as determined by $S_{Property}$, are shown in Figure 4a, alongside the curves for the representative samples within the optimal parameter sets for each filament, as determined by the $S_{Combined}$ values, in Figure 4b; the $S_{Combined}$ values will be reported shortly after. It is worth noting that since many of the samples with the best mechanical properties are also those with the optimal parameter sets, several curves are identical in both Figure 4a,b. In both sets of curves in Figure 4, all filaments have similar E values around 600 MPa, regardless of brand or color. However, significant disparities in $\sigma_{UTS}$ and $\varepsilon_{f}$ exist between filament types, mainly based upon brand. Within each filament color, the SUNLU specimens consistently demonstrate higher $\sigma_{UTS}$ values; however, the SUNLU specimens also exhibit minimal plasticity after reaching their $\sigma_{UTS}$ and have smaller $\varepsilon_{f}$ values compared to the OVERTURE and HATCHBOX samples. This tradeoff of strength and ductility is commonly seen in polymer samples, regardless of whether they are processed through FFF or other 3DP processes [64,65,66]. These differences in tensile properties highlight the diverse behavior that 3D-printed specimens can display by changing filament manufacturer and color, emphasizing further that filament-to-filament variations exist and that 3DP users must take this into account when fabricating components.

Weighing both the dimensional accuracy and mechanical properties through Phase 1 testing, the optimal parameter sets for each filament were determined using the $S_{Combined}$ scores in Table 3. Apart from the HATCHBOX Green filament, the optimal parameter sets for all other filaments were either “Standard” or “80 °C”; the worst parameter sets were scattered depending on filament type, but the “Unheated” condition was most frequent. It appears that these results were mostly driven by mechanical properties, where the “Standard” and “80 °C” bed conditions likely improved the first layer adhesion and subsequent interlayer bonding, thus enhancing mechanical performance to a point where the significance of dimensional accuracy was outweighed compared to other print conditions. However, in several samples, such as the OVERTURE White and SUNLU White samples, specimen geometry was the dominating factor that determined the optimal parameter set. 

The Phase 1 calibrations for the PLA filaments, varying in brand and color, demonstrate that customized printer settings must be made for a specific filament. As filaments vary in processing histories, degree of pigmentation, molecular weights, etc., their response to a given set of printer conditions will likely vary, even if they are categorized as the same material type. Thermal effects on a print’s geometrical accuracy and mechanical properties must be considered during filament calibration steps. With the optimal Phase 1 parameter sets noted as Table 3’s scores for each filament type, further parameter optimization can be completed with adjustments of “slicer” settings, such as infill density and infill pattern, in Phase 2.

### 3.2. Phase 2—“Slicer” Adjustments

After the nozzle temperature and bed conditions are calibrated for a given filament on an FFF printer, processing parameter adjustments can be manipulated in a “slicer” software to produce customized parts with varying properties. For prototyping applications, the two commonly varied parameters are infill density and infill pattern. Infill density is typically adjusted to less than 100% to produce lightweight components, while also saving time and money by using less filament and completing prints in a shorter timeframe. Infill pattern can be customized to provide differing degrees of structural support to a component, particularly if it is utilized in a semi-functional manner where the integrity of mechanical behavior matters. 

In Phase 2, three infill densities—10%, 50%, and 100%—and three infill patterns—Lines, Triangles, and Gyroid—were explored using the Phase 1 optimal parameter sets to observe how mechanical properties and dimensional accuracy changed with processing parameter selection. Similar to Phase I, after tensile testing, the dimensional accuracy and mechanical properties of the samples in each print condition for each filament were ranked, which $S_{Dimension}$ and $S_{Property}$ values are reported in Table 4 after being calculated using Equations (1) and (4), respectively. No apparent trends are observed with the $S_{Dimension}$ values as the infill density or pattern is changed. Any standout values here are likely determined by print-to-print variations, as the prints for each filament using different parameter sets were completed on different days.

While infill settings do not appear to greatly influence print geometry, mechanical properties vary significantly, particularly with infill density. The 100% infill density produced the best tensile properties across all filament types, whereas the 10% infill density typically resulted in the worst properties. Consistent with the literature findings, as infill density is decreased, the void space within the printed samples increases; as these void spaces are often defined by sharp, faceted boundaries, the increased number of stress concentrations leads to lower strength values and worse ductility [22,67]. Interestingly, the worst parameter set for mechanical properties uses the Lines pattern at 10% infill density in all filaments except the HATCHBOX Green, which uses the Triangles pattern at 50% infill density. The angular void spaces created using the sub-100% infill density in these two parameter sets are contrasted by the rounded void spaces produced using the Gyroid infill pattern at all sub-100% infill densities, which reduce the number of stress concentrations and make crack nucleation and propagation more difficult. As such, higher $S_{Property}$ values are seen in nearly all filaments for the Gyroid infills compared to the Lines and Triangles infills at each respective sub-100% infill density. The disparities in infill pattern configurations are visualized using the “slicer” outputs in Figure 2. Future work should include fractography analysis to confirm the influence of stress concentrations on specimen failure mechanisms derived from infill pattern.

**Figure 5:**
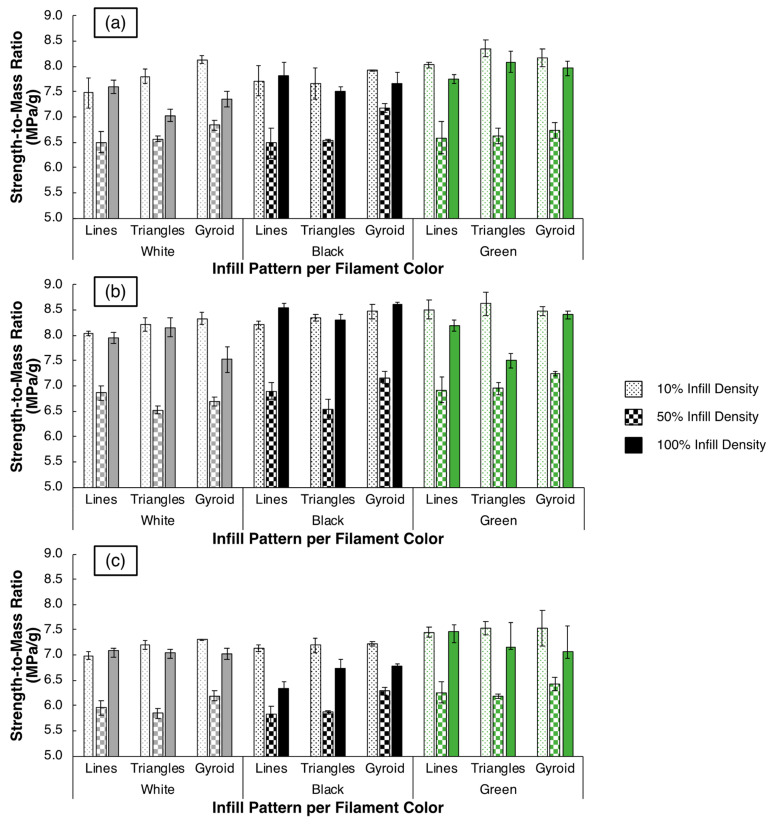
Average strength-to-mass ratio ($\frac{\sigma_{UTS}}{m}$) values at each infill density, infill pattern, and filament color for the (**a**) OVERTURE, (**b**) SUNLU, and (**c**) HATCHBOX specimens with differences in bar color delineating the filament color and differences in the bar pattern delineating the specimen infill density.

While the sub-100% infill density specimens exhibit inferior strength and ductility compared to their 100% infill density counterparts, one must consider the property variation normalized with specimen mass. As lightweight components are often printed at lower infill densities, a calculation of $\frac{\sigma_{UTS}}{m}$, for instance, is warranted for each filament at each parameter set. The average $\frac{\sigma_{UTS}}{m}$ values for the OVERTURE, SUNLU, and HATCHBOX filaments, which are segmented by filament color, infill pattern, and infill density, are plotted in Figure 5a–c, respectively. For every set of prints with a given filament type, filament color, and infill pattern, the 50% infill density appears to produce the worst $\frac{\sigma_{UTS}}{m}$ ratio. There are minimal differences between the values for the 10% and the 100% infill density specimens, yet the $\frac{\sigma_{UTS}}{m}$ values for the 10% infill density specimens usually are higher than the 100% infill density specimens. This indicates that there is a tradeoff when choosing a lower infill density to create semi-functional prints. If users desire higher strength values, they may choose a higher infill density; however, if specimen mass must be considered, caution should be taken to select an appropriate infill density that yields the greatest return on investment. In the case of $\frac{\sigma_{UTS}}{m}$ values, infill pattern and filament color do not appear to make a significant difference. The filament brand, however, appears to influence these values, with lower values corresponding to the 10%, 50%, and 100% infill density samples made using the HATCHBOX filament—at 7.29 ± 0.22 MPa/g, 6.09 ± 0.24 MPa/g, and 6.97 ± 0.37 MPa/g, respectively— across infill patterns and filament colors compared to the OVERTURE filament—at 7.92 ± 0.32 MPa/g, 6.67 ± 0.26 MPa/g, and 7.64 ± 0.34 MPa/g, respectively—and the SUNLU filament—at 8.36 ± 0.21 MPa/g, 6.87 ± 0.27 MPa/g, and 8.14 ± 0.41 MPa/g, respectively. These results demonstrate that 3DP users must consider filament brand in combination with print infill density to maximize $\frac{\sigma_{UTS}}{m}$ values for a given part build.

Combining the scores from Table 4 using Equation (3), the $S_{Combined}$ scores were calculated and reported in Table 5. It is quite evident that mechanical properties are again dominating the selection of optimal parameter sets, as indicated by the lowest $S_{Combined}$ values, where the 100% infill density samples far outperform all other infill densities to such a significant degree that dimensional accuracy plays a minimal role. Infill pattern does not have a consistent effect on the optimal parameter set as identical infills are produced for all patterns at 100% density, as depicted by Figure 2 in Section 3.2. The worst parameter sets in Phase 2, as indicated by the highest $S_{Combined}$ values in Table 5, typically correspond to 10% infill density and either Lines or Triangles as an infill pattern. These parameters are, again, likely driven by the inferior mechanical performance from the higher degree of stress concentrations associated with an increased void space. Using the optimal and worst-case Phase 2 parameter sets indicated by Table 5, the effects of filament thermal characteristics and post-processing were investigated in Phase 3 as a means of improving mechanical performance.

### 3.3. Phase 3—Thermal Analysis and Post-Processing

Annealing, as a post-processing treatment, was investigated with all nine PLA filaments for the optimal and worst-case parameter sets identified in Phase 2 to observe how part geometry and tensile properties vary in ideal and non-ideal print scenarios. Before selecting the annealing conditions, the thermal characteristics of all PLA filaments must first be considered so that proper treatment times and temperatures might be selected. 

**Figure 6:**
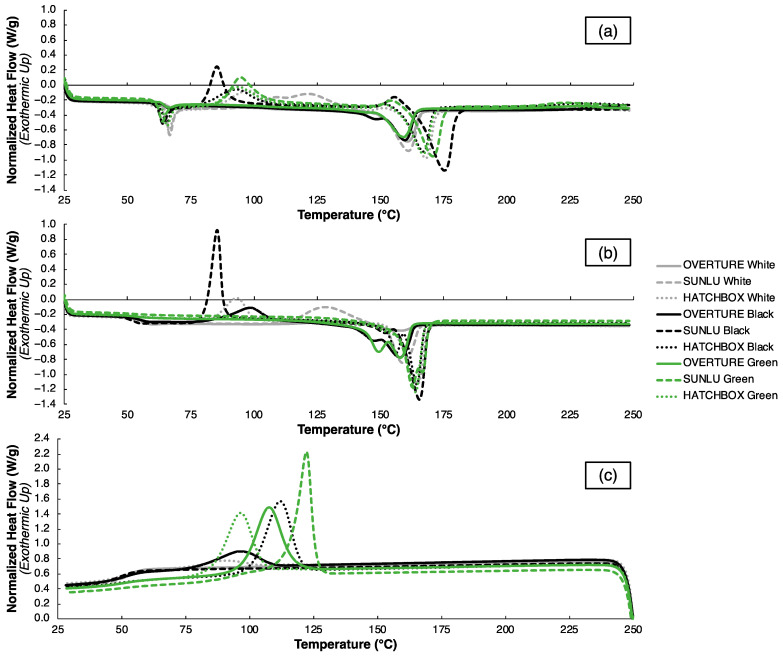
DSC curves for all nine filaments upon heating in the (**a**) as-fabricated and (**b**) normalized states from DSC Runs #1 and #2, respectively, and (**c**) upon cooling between Runs #1 and #2.

The DSC curves in Figure 6 reveal the thermophysical transitions upon heating and cooling for all nine filaments, where the normalized heat flow (in W/g) is plotted against temperature (in °C) for the filaments in the as-fabricated state upon heating for DSC Run #1 in Figure 6a, in the normalized state upon heating for DSC Run #2 in Figure 6b, and upon cooling between Runs #1 and #2 in Figure 6c. Specific values extracted from the DSC heating curves in Figure 6a,b are reported in Table 6. The data from the as-fabricated state are useful to describe the effects of processing history, typically from filament production, on the thermal behavior of the PLA filaments, which resembles the condition of the filament prior to melting in FFF. On the other hand, the data from the normalized state—which are generated upon melting after heating the as-fabricated filament in Figure 6a—are useful to describe the thermal behavior of PLA independent of its fabrication method, which may better describe the nature of the material after melting in FFF. In Figure 6a, three distinct transitions are observed: (1) glass transition, (2) cold crystallization, and (3) melting. Similar glass transitions are observed for all filaments, with an average T_g_ at 64.67 ± 1.27 °C, which is typical for PLA, as indicated by an exaggerated endothermic peak upon heating in Figure 6a. This endothermic peak at the glass transition is indicative of residual stresses from prior thermal processing—in this case, filament fabrication—which are relieved due to molecular relaxation upon heating [68]. Another by-product of residual stresses is the shifting of the T_g_ values when compared to the stress-free polymer; this will be compared shortly to the stress-free PLA in the normalized state in Figure 6b. Similar melting behavior across all filaments is also seen, with an average T_m_ of 165.41 ± 5.56 °C, as indicated by the endothermic peak expected for semi-crystalline PLA. The melting peaks appear to vary over a much larger range compared to the glass transition, with the lowest peak temperatures for the OVERTURE filaments and the highest peak temperatures for the SUNLU filaments. The same pattern is seen with the $\Delta H_{m}$ values, as indicated by the area under the curve in Figure 6a upon melting, where $\Delta H_{m}$ represents the energy released during the melting of crystalline regions in a polymer. It is important to note that the T_g_ and T_m_ values for the nine filaments considered are related to the recommended FFF print bed and nozzle temperatures for PLA, respectively. Specifically, the T_g_ and T_m_ values here are slightly lower than the ideal bed and nozzle temperatures, indicating that slightly higher values are ideal for FFF than the peak temperatures observed through DSC. If known, 3DP users can make more informed processing parameter selection using thermal characterization data to achieve successful filament melting and part production.

Cold crystallization behavior is far more variable in the filaments compared to the glass transition and melting behaviors, as observed in the exothermic peaks between the T_g_ and T_m_ in Figure 6 and the respective values in Table 6. Where applicable, the average peak cold crystallization temperature (T_cc_) value across all filaments is 97.77 ± 12.38 °C, which is consistent for the HATCHBOX filaments (94.95 ± 2.2 °C) and relatively spread out for the SUNLU filaments (100.58 ± 18.82 °C), ranging from 85.30 °C (SUNLU Black) to 131.61 °C (SUNLU White); no OVERTURE filaments exhibit cold crystallization tendencies, as indicated by “N/A” in Table 6. Thermal history and additive content are major factors influencing cold crystallization behaviors, given the reliance of crystallization kinetics upon the nucleation sites present in the original filament melt [47,69]. Given the previously observed differences between the as-fabricated filaments, it is not surprising to see this variation. The disparities in cold crystallization across the filament types are echoed by the variations in χ, mainly by brand, as seen in Table 6. Crystallinity values, as calculated by Equation (5), range from 6.62% (SUNLU Green) to 26.25% (OVERTURE Green) from Run #1. Typically, this spread in χ would be justified by observing the baseline shift at the glass transition, which is reflected by the ∆C_p_ values, as reported in Table 6. Given that glass transition only applies to the amorphous regions of a polymer, smaller ∆C_p_ values—and, therefore, a less pronounced glass transition—are observed if the amorphous content is lower, thus indicating a higher χ [70]. However, from the Run #1 data in Table 6, this trend is not clear. This is likely due to the fact that the residual stress state of the as-fabricated PLA filaments is high from thermomechanical processing during filament production, which has been reported to skew DSC data without normalization [68,71]. While studying cold crystallization and χ is interesting from an academic perspective, this is less relevant in a practical 3DP scenario, as the PLA filaments will be melted anyway in their as-fabricated state through the heated nozzle. As such, these trends are most relevant to observe with the Run #2 data after normalization, which most closely resemble the characteristics of the printed component.

Several distinct differences can be observed in the nine PLA filaments after normalization from Run #2 DSC data in Figure 6b and Table 6. A tight spread is still observed in the T_g_ values, averaging 54.39 ± 1.78 °C. These values are shifted approximately 10 °C to lower temperatures compared to Run #1; additionally, the severe endothermic peak during the glass transition from Run #1 is not observed; instead, there is a gradual baseline shift that is expected during the glass transition. This is logical given that residual stress alleviation is typically accompanied by lowered T_g_ values and an absence of endothermic peaks during the glass transition when analyzed through DSC, as previously mentioned. Similar T_m_ values are observed from Run #2 data compared to Run #1, with the characteristic endothermic peak at several degrees lower than Run #1, and an average of 161.50 ± 3.26 °C. For most filaments, the $\Delta H_{m}$ values appear to increase for the second run; the exceptions here are the OVERTURE White and SUNLU White filaments. This is characteristic of increased crystallinity typically observed in normalized filaments. The cold crystallization behavior is equally as variable in Run #2 compared to Run #1, with several filaments exhibiting crystallization that was not observed in Run #1 and several filaments not showing crystallization that was observed in Run #1. The T_cc_ values average to be 103.69 ± 16.94 °C in Run #2, when applicable, with values ranging from 85.42 °C (SUNLU Black) to 127.53 °C (SUNLU White). Even with normalization from the first melt, cold crystallization relies heavily on molecular weight, stereochemistry, etc., so it is not surprising to see these variations repeated in Run #2. The χ values also fluctuate, ranging from 0.94% (OVERTURE White) to 58.49% (SUNLU Green). In this case, however, crystallinity does not rely upon the original manufacturing method but, instead, varies with the cold crystallization behavior, which is derived from how the PLA filaments have responded to the cooling step after melting in Run #1. Interestingly, all filaments that exhibit a cold crystallization peak upon heating in Run #2 do not exhibit an appreciable exothermic crystallization peak upon cooling between Runs #1 and #2, as shown in Figure 6c. This is likely due to each filament’s tendency to nucleate crystals in the melt upon cooling at the specified cooling rate (10 °C/min), which is influenced by factors such as polymer composition and molecular weight [72]. 

The DSC data analysis from Figure 6 and Table 6 highlights distinct thermal behaviors for the nine PLA filaments, which are reliant upon thermal history and molecular differences induced by manufacturing. The trends in thermophysical tendencies upon heating and cooling revealed from the DSC analysis are useful in a 3DP environment, as thermal cycles are often encountered that fluctuate between a solid state and a molten state, which rely upon the filaments’ thermal characteristics. The Run #1 heating data, particularly the T_g_ and T_m_, are indicative of the filament going into the FFF printer and can inform the selection of proper print bed and nozzle temperatures, respectively, to achieve adequate melting and bonding during extrusion. The Run #2 data, on the other hand, are more indicative of what can be expected in the printed component, as they take into account a normalization step from melting. However, the cooling rates and thermal cycles involved with FFF are not accounted for in the cooling rates between Runs #1 and #2, nor the heating rates in Run #2; thus, variations are expected to occur between practical FFF processing and the ideal DSC environment. With an understanding of the thermal characteristics of the as-fabricated and normalized PLA filaments, a proper annealing treatment can be selected. Recommended annealing conditions for PLA vary depending on part geometry, but samples are typically processed between the T_g_ and T_m_ to provide enough energy to kickstart crystallization without creating dimensional instabilities. For FFF-printed PLA specimens, annealing times vary quite substantially in the literature from as little as 5 min to as long as 2 h [55,73,74]. In this study, an annealing temperature of 75 °C was chosen, which is just above the normalized filaments’ average T_g_ value. Annealing was performed for 15 min after the samples reached 75 °C, which is on the lower end of the processing times observed in the literature. A shorter annealing period was explored in hopes of minimizing part distortion, given that the specimens were not fixtured in a mold during annealing and were, therefore, more susceptible to geometrical changes with longer treatment times.

Before identifying mechanical property changes with annealing, the effects of post-processing on specimen dimensions should be discussed. Table 7 highlights the percent differences in tensile specimen geometry, according to Figure 2 labeling, between the as-printed and annealed conditions for each filament using both the optimal and worst-case Phase 3 parameter sets. For nearly all specimens, the specimen size decreases in the XY-plane and increases in the Z-direction after annealing; specifically, dimensions A through E typically decrease, while dimension F increases. Dimensional changes are not uncommon in polymer annealing and are typically associated with residual stress relief and crystallization [51,75]. Large percent changes, however, were observed in several samples—as high as 6.57%—which is undesirable for prints that require tight tolerances. Specimen fixturing, such as in a mold, or different annealing conditions may be required to mitigate the effect of dimensional changes during post-processing. 

Changes in mechanical properties after post-processing were also tracked, and the percent differences in key tensile properties between the annealed and as-printed conditions for each filament for both the optimal and worst-case parameter sets are reported in Table 8. There appears to be substantial variation in the magnitude of mechanical property changes after post-processing for each filament brand, color, and parameter set. The values of $\frac{\sigma_{UTS}}{m}$ change most consistently, with the values being nearly all lower in the annealed state compared to the as-printed state. These results are not ideal for an annealed condition, as increases in strength, and sometimes ductility, are expected with proper thermal processing. All other variations in stiffness, strength, and ductility metrics, which seemingly scatter across filament types, are likely associated with a non-optimized set of treatment conditions for the different filaments, further indicating the need for material-specific post-processing optimization to improve part properties.

**Figure 7:**
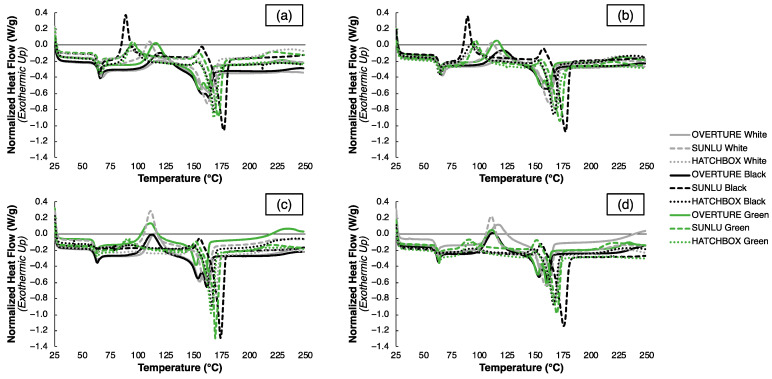
DSC curves upon heating for the optimal and worst-case parameter sets in the as-printed condition ((**a**) and (**b**), respectively) and the annealed condition ((**c**) and (**d**), respectively).

The variable mechanical property changes with annealing are reflected by similar trends in the printed specimens’ thermal characteristics, as seen in the DSC curves of both the as-printed and annealed samples using the optimal and worst-case parameter sets in Figure 7, and the changes in T_g_, T_cc_, and T_m_ after annealing for both parameter sets in Table 9; the tabulated DSC data used to calculate the values in Table 9 are reported in Spreadsheet S9 (“Printed Specimen DSC Tabulated Values”) as Supplementary Materials. Minimal differences can be seen between the curves of the as-printed specimens from the optimal and worst-case parameter sets in Figure 7a,b; however, larger differences are notable between the curves of the samples in the annealed condition in Figure 7c,d. This could be justified based upon the thermal gradients imparted to the samples during annealing for a specific parameter set, given that the samples from the worst-case parameter sets had less than 100% infill density, whereas the samples from the optimal parameter sets all had 100% infill density. With a higher void space content and a lower mass in a given sample for the worst-case printed samples, heat-transfer kinetics occur more rapidly during annealing and may cause different thermophysical transitions in the printed specimens. This is reflected in the different residual stress states, variable χ values, and changes in peak characteristics in the annealed specimens from the worst-case parameter sets versus the optimal parameter sets.

Comparing the as-printed and annealed samples from the optimal parameter sets in Figure 7a,c, respectively, and from the worst-case parameter sets in Figure 7b,d, respectively, large deviations in peak positions are observed. Table 9 highlights the extent of these changes, most notably with an average decrease in T_g_ and T_m_ for nearly all samples upon annealing. This is reflected by the marginally smoother glass transitions and more pronounced melting peaks in the associated regions of Figure 7c,d. The T_cc_ values vary more substantially, though, for all samples after post-processing, either increasing or decreasing depending on the sample. This is evident through the changes in peak positions on the DSC curves in Figure 7c,d. The decreases in the T_g_ and T_m_ are characteristic of residual stress relief, which is expected upon annealing. However, the variation in T_cc_, as well as the χ values in Table 9, demonstrates differing degrees of efficacy of the annealing treatment, depending on the sample. Specifically, annealing is expected to increase the degree of crystallinity in the semi-crystalline polymer by providing enough energy to create short- or long-range order; yet, in the samples considered herein, some specimens decrease χ substantially after annealing. This indicates non-ideal annealing conditions at 75 °C and 15 min for certain filaments, whether it be non-ideal temperature or time, while these conditions are satisfactory for other filaments. Additional optimization of post-processing conditions per filament type and processing parameter set would be necessary to achieve ideal annealing, further indicating how differing filaments require specific optimization steps in 3DP, even if they belong to the same material type and are printed in similar geometries.

### 3.4. Roadmap for FFF Filament Processing Optimization

Printer calibrations, “slicer” setting adjustments, and thermal analysis coupled with annealing in Phases 1 through 3, respectively, yield notable outcomes when processing PLA in FFF. The Phase 1 print calibrations highlight the importance of fine-tuning nozzle and bed temperatures, as well as bed surface, to achieve adequate layer adhesion, high-fidelity prints, and subsequent control of specimen geometry and mechanical performance. These calibration results are highly dependent upon filament brand and color, aligning with the deviations in thermal characteristics in the Phase 3 DSC analysis. The Phase 2 testing reveals how dimensional accuracy and tensile behavior can be affected by systematic “slicer” setting modifications, all reliant upon filament characteristics and desired 3D-printed part specifications. Annealing also produces notable discrepancies in dimensional and mechanical outputs as filament type changes.

With the filaments utilized in this study, an abundance of evidence indicates the need for making FFF processing decisions with filament characteristics in mind. If these are ignored, improper instrument settings will lead to sub-par component quality and increased waste of time, money, and resources. Furthermore, the process optimization steps outlined in Phase 1 through Phase 3 should be followed in the specified order. As all steps of the FFF process are interconnected, non-ideal temperature settings in nozzle calibration, for instance, may result in reduced mechanical integrity through tensile testing, leading to inferior performance after post-processing than could have otherwise been achieved. While these claims can only be confidently purported in the context of the FFF-printed PLA specimens used in this study, these filament-to-filament variations are likely to have downstream processing effects in FFF with other material types. In fact, these differences may be more pronounced with difficult-to-process materials, such as ABS and newer composite blends. Therefore, the framework of FFF process optimization established in this study using PLA—defined by printer calibrations followed by “slicer” setting adjustments and finally post-processing—should be used to produce the highest-quality components across all filament types, balancing mechanical properties and dimensional accuracy for the desired application.

## 4. Conclusions

In this study, the effects of processing parameters and printer conditions on the dimensional accuracy and mechanical properties of FFF-printed PLA specimens were investigated. Using nine commercially available filaments that vary in brand and color, the following conclusions could be made:

- The Phase 1 print calibrations revealed different suitable nozzle temperatures and print bed conditions depending on the filament type. Nozzle temperatures appeared to be at the upper end of manufacturer recommendations, with color and brand both influencing the optimal values. Proper selection of bed temperature and surface preparation may serve to decrease thermal gradients in the printed component and enhance layer adhesion, resulting in mitigated deviation from the prescribed specimen geometry and improved tensile behavior. Specimen $\sigma_{UTS}$ and $\varepsilon_{f}$ values appear to diverge most substantially based on filament brand, likely due to the effects of filament fabrication using different manufacturing processes and chemical formulations. Nozzle temperature and bed conditions should be optimized on a per-filament basis, even if multiple filaments are categorized as the same material type.
- Modifications to infill pattern and infill density in Phase 2 significantly impacted 3D-printed specimen mechanical properties, whereas specimen geometry was minimally affected. Infill density appeared to produce the most pronounced changes in tensile behavior, with a precipitous drop-off in strength and ductility at sub-100% infill density. Infill pattern was most influential in non-fully dense specimens, where higher stress concentrations in the sharp, faceted Lines and Triangles patterns caused a reduction in mechanical integrity compared to the smooth, rounded Gyroid pattern. Trends in $\frac{\sigma_{UTS}}{m}$ ratios add complexity to infill setting selection, as an increase in $\frac{\sigma_{UTS}}{m}$ was observed when infill density was reduced from 50% to 10%, with notable differences from brand to brand as well. 3DP users should consider the ramifications of print time and material usage based on the combination of infill density and infill pattern to achieve the desired balance of mechanical properties for the printed specimen’s end use.
- The DSC analysis and annealing treatments in Phase 3 highlighted fundamental disparities in the thermal characteristics between the filaments. The T_g_ and T_m_ values of the filaments in the as-fabricated state emphasized the effect of thermal history and residual stresses on thermophysical behavior. These conditions also aligned with recommended print bed and nozzle temperatures, respectively, for the PLA filaments. The normalized DSC runs provided a better indication of the thermal characteristics of the 3D-printed specimens, given the similar melting step during both DSC normalization and FFF material extrusion. Annealing at 75 °C for 15 min resulted in large changes in specimen geometry and increased scatter in mechanical property values. Part shrinkage in the XY-plane, accompanied by an increase in specimen thickness, at levels higher than desired was observed after annealing. Fluctuations in all mechanical property metrics after annealing revealed the varying degree of treatment efficacy based upon filament type, which was confirmed by the equally scattered DSC data, thus necessitating filament-specific post-processing conditions for ideal geometrical and mechanical property outcomes. Infill density should also be considered during annealing time and temperature selection, as heat-transfer kinetics will vary depending on part mass and void content.

By implementing the multi-step framework for FFF process optimization in this study, notable effects of processing parameters on part quality and behavior were observed across the PLA filament types. As FFF relies upon the feedstock material’s thermophysical characteristics, filament-specific processing parameter selection should be made, even if separate filaments consist of the same material type. By making more informed processing decisions, which are supported by material characterization data, 3DP users can apply this optimization strategy to all feedstock material types, leading to improved material processability and printed part performance in functional FFF applications.

## Figures and Tables

**Table 1:** Optimal nozzle temperatures for each brand–color combination qualitatively determined using temperature towers.

	OVERTURE	SUNLU	HATCHBOX
White	205 °C	220 °C	205 °C
Black	215 °C	230 °C	210 °C
Green	220 °C	220 °C	215 °C

**Table 2:** Filament scoring across all four printer conditions, with varying bed temperature and bed surface, based upon dimensional accuracy and mechanical properties ($S_{Dimension}$ and $S_{Property}$ separated as $S_{Dimension}\;/\;S_{Property}$).

		“Standard”	“Unheated”	“80 °C”	“Glass”
White	OVERTURE	20/33	34/39	34/21	32/27
	SUNLU	20/21	36/33	44/21	20/45
	HATCHBOX	32/36	34/30	38/15	16/39
Black	OVERTURE	30/30	28/48	30/15	32/27
	SUNLU	32/12	36/33	20/45	32/30
	HATCHBOX	24/21	32/21	40/42	24/36
Green	OVERTURE	28/42	30/33	32/18	30/27
	SUNLU	28/30	36/27	30/21	26/42
	HATCHBOX	24/33	34/27	36/45	26/15

**Table 3:** Filament scoring across all four printer conditions, with varying bed temperature and bed surface, produces $S_{Combined}$ values based upon the contributions from dimensional accuracy and mechanical properties (* optimal parameter set identified for a given filament).

		“Standard”	“Unheated”	“80 °C”	“Glass”
White	OVERTURE	53 *	73	55	59
	SUNLU	41 *	69	65	65
	HATCHBOX	68	64	53 *	55
Black	OVERTURE	60	76	45 *	59
	SUNLU	44 *	69	65	62
	HATCHBOX	45 *	53	82	60
Green	OVERTURE	70	63	50 *	57
	SUNLU	58	63	51 *	68
	HATCHBOX	57	61	81	41 *

**Table 4:** Filament scoring across all nine printer conditions, with varying infill density and infill pattern, based upon dimensional accuracy and mechanical properties ($S_{Dimension}$ and $S_{Property}$ separated as $S_{Dimension}/S_{Property}$).

		Lines			Triangles			Gyroid		
		10%	50%	100%	10%	50%	100%	10%	50%	100%
White	OVERTURE	205/216	145/198	145/48	160/204	150/192	200/114	80/150	110/156	155/72
	SUNLU	190/210	125/144	170/72	100/192	145/210	225/48	75/186	145/180	150/108
	HATCHBOX	170/228	200/168	125/42	205/204	125/210	115/84	175/180	100/144	135/90
Black	OVERTURE	155/222	130/180	110/48	170/222	120/198	200/96	150/162	155/150	160/72
	SUNLU	150/228	115/180	205/48	105/210	120/204	115/84	165/192	155/144	190/60
	HATCHBOX	160/210	75/150	165/108	120/204	160/210	175/96	160/180	190/138	120/54
Green	OVERTURE	105/216	140/180	205/90	100/180	85/186	190/60	165/192	205/168	150/78
	SUNLU	235/210	160/186	120/84	195/182	75/182	135/90	190/192	135/144	95/60
	HATCHBOX	155/198	120/162	165/42	125/186	55/216	170/84	175/204	155/150	225/102

**Table 5:** Filament scoring across all nine printer conditions, with varying infill density and infill pattern, produces $S_{Combined}$ scores based upon the contributions from dimensional accuracy and mechanical properties (* optimal parameter set identified for a given filament; ** worst-case parameter set identified for a given filament.)

		Lines			Triangles			Gyroid		
		10%	50%	100%	10%	50%	100%	10%	50%	100%
White	OVERTURE	421 **	343	193 *	364	342	314	230	266	227
	SUNLU	400 **	269	242 *	292	355	273	261	325	258
	HATCHBOX	398	368	167 *	409 **	355	199	355	244	225
Black	OVERTURE	377	310	158 *	392 **	318	296	312	305	232
	SUNLU	378 **	295	253	315	324	199 *	357	299	250
	HATCHBOX	370 **	225	273	324	370 **	271	340	328	174 *
Green	OVERTURE	321	320	295	280	271	250	357	373 **	228 *
	SUNLU	455 **	346	204	387	267	225	382	279	155 *
	HATCHBOX	353	282	207 *	311	271	254	379 **	305	327

**Table 6:** Extracted values from the DSC Runs #1 and #2 for all nine filaments, where “N/A” indicates that no value was measured for the given filament.

	T_g_ (°C)		∆C_p_ at Glass Transition (J/g°C)		T_cc_ (°C)		$\Delta H_{cc}\;(J/g)$		T_m_ (°C)		$\Delta H_{m}\;(\text{°}C)$		χ (%)	
	Run #1	Run #2	Run #1	Run #2	Run #1	Run #2	Run #1	Run #2	Run #1	Run #2	Run #1	Run #2	Run #1	Run #2
OVERTURE White	66.57	57.02	0.184	0.465	N/A	113.94	N/A	5.26	160.78	158.32	22.86	4.37	24.42	0.94
SUNLU White	65.89	56.77	0.491	0.485	121.61	127.54	27.49	21.16	160.99	158.82	35.89	31.22	8.97	10.75
HATCHBOX White	65.46	55.16	0.648	0.445	97.48	93.03	19.25	20.00	167.87	164.61	29.12	35.87	10.54	16.96
OVERTURE Black	65.17	54.78	0.157	0.393	N/A	98.54	N/A	16.73	159.88	157.67	20.84	35.97	22.26	20.56
SUNLU Black	62.91	51.95	0.696	0.470	85.30	85.42	23.25	34.33	175.31	165.37	43.17	46.97	21.28	13.50
HATCHBOX Black	64.32	54.00	0.636	0.123	93.48	N/A	18.26	N/A	166.96	164.40	28.22	41.84	10.64	44.70
OVERTURE Green	64.44	54.47	0.244	0.170	N/A	N/A	N/A	N/A	159.22	157.89	24.57	39.80	26.25	42.52
SUNLU Green	62.74	53.03	0.547	0.158	94.84	N/A	26.97	N/A	170.81	162.61	33.17	54.75	6.62	58.49
HATCHBOX Green	64.51	52.35	0.598	0.221	93.90	N/A	19.97	N/A	166.84	163.84	29.56	42.39	10.25	45.29

**Table 7:** Percent differences in the tensile specimen dimensions (with original units provided) between the annealed and as-printed conditions for each filament for both the optimal and worst-case parameter sets.

		Parameter Set	A (mm)	B (mm)	C (mm)	D (mm)	E (mm)	F (mm)
OVERTURE	White	Optimal	−4.00%	−4.15%	−4.83%	−2.40%	−5.69%	6.57%
		Worst-Case	−3.94%	−3.24%	−4.64%	−4.49%	0.85%	5.55%
	Black	Optimal	−1.23%	−1.43%	−1.62%	−2.46%	−0.33%	2.34%
		Worst-Case	−0.88%	−2.14%	−1.92%	−3.07%	0.51%	1.57%
	Green	Optimal	−0.65%	−0.31%	−1.67%	−2.01%	1.84%	4.39%
		Worst-Case	−0.61%	−0.50%	−1.26%	−4.01%	3.64%	3.78%
SUNLU	White	Optimal	−2.29%	−2.26%	−2.52%	−1.70%	−2.67%	1.80%
		Worst-Case	−2.01%	−2.26%	−2.55%	−2.79%	0.17%	2.09%
	Black	Optimal	−0.47%	−0.65%	−1.15%	−1.09%	0.33%	3.19%
		Worst-Case	−0.65%	−0.74%	−1.56%	−1.70%	0.45%	3.10%
	Green	Optimal	−0.69%	−0.46%	−0.26%	0.06%	−0.73%	0.60%
		Worst-Case	−0.57%	−0.99%	−0.45%	−0.02%	−0.91%	0.32%
HATCHBOX	White	Optimal	−0.51%	−0.16%	−0.60%	0.29%	−2.29%	−1.34%
		Worst-Case	−0.54%	−0.85%	−0.54%	−0.54%	0.79%	0.14%
	Black	Optimal	−2.00%	−2.05%	−1.74%	−2.48%	−1.67%	1.76%
		Worst-Case	−1.95%	−2.35%	−2.15%	−2.69%	−0.11%	3.21%
	Green	Optimal	−1.08%	−1.39%	−2.13%	−1.86%	−0.06%	0.75%
		Worst-Case	−1.60%	−1.69%	−1.53%	−1.84%	1.53%	2.01%

**Table 8:** Percent differences in mechanical properties (with original units provided) between the annealed and as-printed tensile specimens for each filament for both the optimal and worst-case parameter sets.

		Parameter Set	E (MPa)	$\sigma_{y}\;(\mathrm{MPa})$	$\sigma_{UTS}\;(\mathrm{MPa})$	$\varepsilon_{f}\;(\%)$	$\frac{\sigma_{UTS}}{m}\;(\mathrm{MPa}/g)$
OVERTURE	White	Optimal	−3.51%	−6.34%	−6.5%	−13.39%	−6.61%
		Worst-Case	10.52%	12.71%	4.53%	−8.8%	−2.59%
	Black	Optimal	−8.4%	−7.41%	−9.79%	−9.19%	−9.9%
		Worst-Case	−18.69%	−1.22%	−2.67%	31.81%	−3.93%
	Green	Optimal	−7.51%	0.24%	−2.21%	18.34%	−4.82%
		Worst-Case	−13.21%	2.46%	1.56%	35.24%	−2.39%
SUNLU	White	Optimal	1.51%	−10.46%	−11.93%	−15.11%	−8.64%
		Worst-Case	0.55%	−2.09%	2.31%	−0.44%	0.02%
	Black	Optimal	−3.85%	5.11%	5.8%	7.97%	3.58%
		Worst-Case	5.33%	8.72%	9.64%	−4.56%	1.58%
	Green	Optimal	−9.97%	−17.78%	−16.78%	−11.22%	−13.2%
		Worst-Case	−11.45%	−10.14%	−11.08%	−1.12%	−5.69%
HATCHBOX	White	Optimal	4.15%	−7.69%	−7.64%	8.38%	−5.74%
		Worst-Case	−0.08%	−4.18%	−5.93%	2.86%	−5.35%
	Black	Optimal	3.58%	−4.96%	−7.15%	−10.15%	−6.24%
		Worst-Case	−7.44%	−1.03%	−1.56%	66.91%	−2.53%
	Green	Optimal	−6.6%	1.78%	−1.85%	−5.47%	−0.48%
		Worst-Case	6.97%	11.61%	6.49%	3.98%	2.15%

**Table 9:** Percent differences in thermal characteristics (with original units provided) between the annealed and as-printed specimens for each filament for both the optimal and worst-case parameter sets.

		Parameter Set	χ (%)	T_g_ (°C)	T_cc_ (°C)	T_m_ (°C)
OVERTURE	White	Optimal	−86.52%	−6.50%	−3.25%	−0.52%
		Worst-Case	37.66%	−6.56%	−8.36%	−0.26%
	Black	Optimal	−76.48%	−5.36%	0.95%	−1.80%
		Worst-Case	−28.91%	−5.45%	−0.40%	−0.02%
	Green	Optimal	33.97%	−5.10%	0.73%	0.97%
		Worst-Case	72.36%	−7.16%	−1.17%	−0.25%
SUNLU	White	Optimal	44.39%	−4.53%	−6.61%	−0.99%
		Worst-Case	−85.29%	−2.45%	−6.83%	−0.40%
	Black	Optimal	128.82%	−5.45%	7.33%	−1.68%
		Worst-Case	129.96%	−5.24%	7.60%	−0.94%
	Green	Optimal	144.14%	−4.62%	−2.67%	−1.15%
		Worst-Case	123.31%	−3.38%	0.22%	−0.10%
HATCHBOX	White	Optimal	21.85%	−2.39%	−4.36%	3.65%
		Worst-Case	−88.56%	2.39%	−3.51%	−1.31%
	Black	Optimal	38.11%	−2.46%	−4.65%	−1.55%
		Worst-Case	85.72%	−2.00%	−5.60%	−1.41%
	Green	Optimal	78.79%	−4.34%	−4.71%	−1.44%
		Worst-Case	86.01%	−1.51%	−3.85%	−0.89%

## Data Availability

The data presented in this study are available in the Supplementary Materials.

## Supplementary Materials

The following supporting information can be downloaded at https://www.mdpi.com/article/10.3390/polym15081945/s1. Spreadsheet S1: Phase 1 Stress–Strain Curves; Spreadsheet S2: Phase 1 Testing Values; Spreadsheet S3: Phase 2 Testing Values; Spreadsheet S4: Phase 3 Testing Values; Spreadsheet S5: Phase 1 Rankings; Spreadsheet S6: Phase 2 Rankings; Spreadsheet S7: Filament DSC Heating and Cooling Curves; Spreadsheet S8: Printed Specimen DSC Heating Curves; Spreadsheet S9: Printed Specimen DSC Tabulated Values.
